# Choroidal metastasis as a presenting feature in a metastatic lung carcinoma

**DOI:** 10.3205/oc000200

**Published:** 2022-05-20

**Authors:** Mukesh Jain, Deepika C. Parameswarappa

**Affiliations:** 1 Anant Bajaj Retina Institute, MTC Campus, L V Prasad Eye Institute, Bhubaneswar, India; 2 Anant Bajaj Retina Institute, KAR Campus, L V Prasad Eye Institute, Hyderabad, India

**Keywords:** choroidal metastasis, lung carcinoma, multi-modal imaging

## Abstract

We report a case of a 65-year-old female who presented to us with diminution of vision in the right eye. She was only able to perceive light in the right eye, and the left eye had a vision of 20/20, N6. Anterior segment examination in both eyes was unremarkable except for senile cataract in the left eye. Posterior segment examination revealed features of choroidal metastasis in both eyes and exudative retinal detachment in the right eye. Multimodal imaging helped in the further confirmation of metastatic lesions. Right-eye fundus autofluorescence showed hyperautofluorescent lesions, ultrasound B-scan showed an elevated mass lesion in the choroid with moderate to high internal echogenicity, and optical coherence tomography showed a lumpy-bumpy appearance of the retinal pigment epithelium as well as an elevated choroidal mass lesion beneath it. On detailed systemic evaluation, the primary site of cancer was found to be the lungs. The patient was referred to a pulmonologist and an oncologist for chemotherapy and further management.

## Background

The choroid is the most common site for ocular metastasis from a systemic carcinoma due to its rich blood supply. The two most common sites of primary causes for ocular metastasis are the breast followed by the lung [1], [2]. The incidence of choroidal metastasis is reported to be 7% from the lung [3]. The patient can present with blurred vision, floaters, flashes, and defective field of vision [4]. A good clinical suspicion with thorough systemic evaluation is needed to diagnose ocular metastasis. In our case, the patient was detected for the first time with systemic malignancy on the basis of her ocular metastatic features. We would like to highlight the important role of the ophthalmologist in diagnosing a life-threatening primary malignancy. Early detection of any malignancy will add to the quality of life years of the patients. We are writing this case report to emphasize the fact that the eye is a window to systemic diseases. 

## Case description

**Figure 1:**
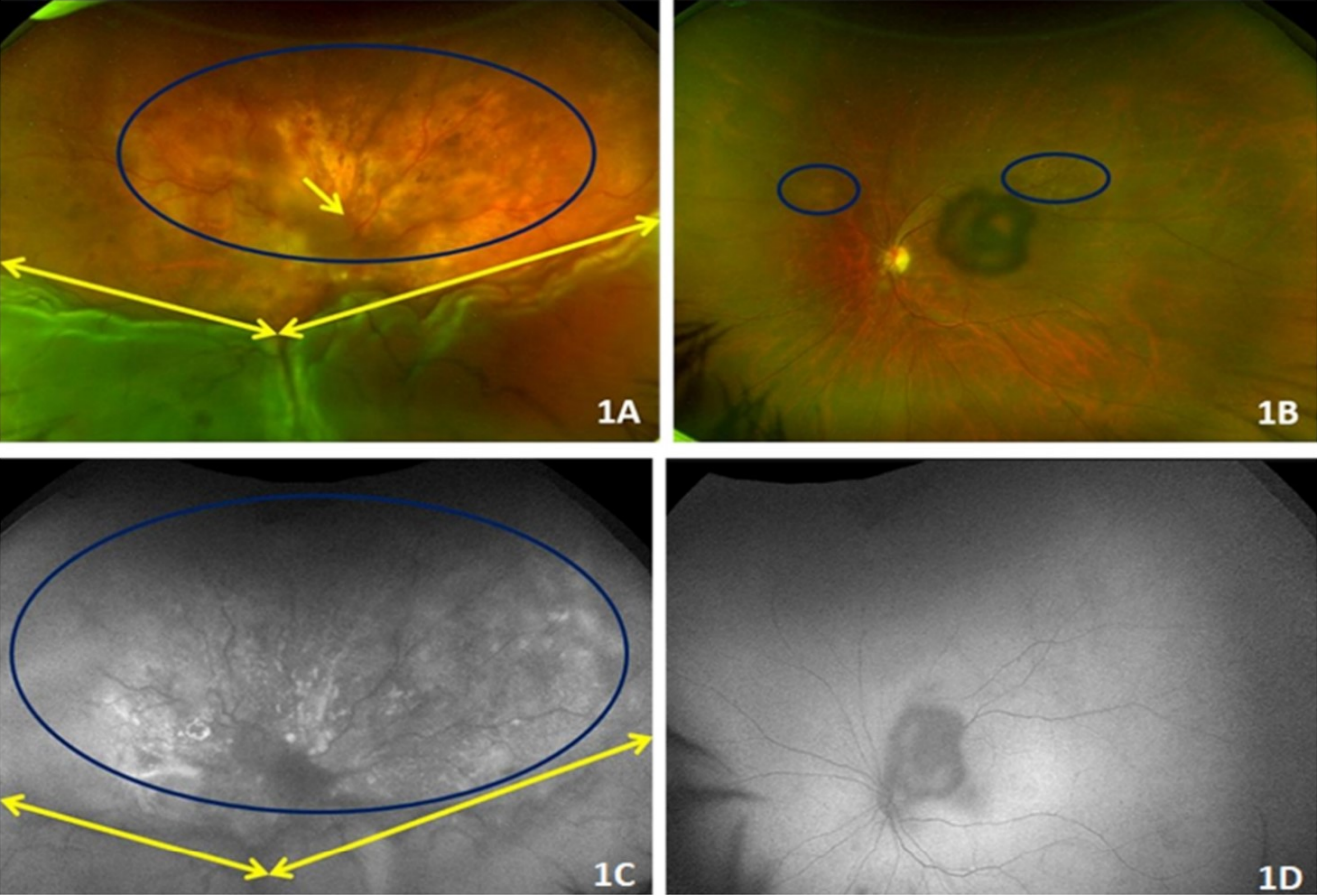
A) Right-eye color fundus photo showed a hazy optic disc, minimal tortuosity of veins, exudative retinal detachment from 3 to 9 clock hours (yellow double-headed arrows), a small pocket of subretinal fluid superior to the optic disc (yellow arrow), and discrete yellowish choroidal lesions with ill-defined margins in the superior quadrant of fundus (blue ellipse). B) Left-eye color fundus showed normal optic disc and vasculature with discrete yellowish choroidal lesions along the superior arcade and superonasal to the optic disc (two blue ellipses), with a central black shadow artifact due to cataract. C) Right-eye fundus autofluorescence showed a mottled hyper- and hypo-autofluorescent pattern in the superior quadrant. D) Left-eye fundus autofluorescence was normal with cataract shadow artifact at the center.

A 65-year-old female presented to us with complaints of blurring of vision in the right eye since two months. She was a known case of hypertension and goiter, and was on treatment for the same. On examination, she was only able to perceive light in the right eye with an inaccurate response to projection of rays. Her left eye had a best corrected visual acuity (BCVA) of 20/20 and N6. Anterior segment examination and intraocular pressure were normal in both eyes except for an early posterior subcapsular cataract in the left eye. The right eye had a clear media with a hazy view of the optic disc, minimal tortuosity of veins, exudative retinal detachment from 3 to 9 clock hours, and a small pocket of subretinal fluid superior to the optic disc. Right-eye fundus showed discrete yellowish choroidal lesions with ill-defined elevated margins in the superior quadrant of fundus with overlying retinal pigment epithelium changes (Figure 1A (Fig. 1)). Left-eye fundus showed normal optic disc and vasculature with a shallow elevated yellowish choroidal lesion along the superior arcade and superonasal to the optic disc with no SRF clinically (Figure 1B (Fig. 1)).

### Investigations

#### Fundus autofluorescence (FAF)

Right-eye fundus autofluorescence showed mottled hyper and hypo autofluorescent pattern in the superior quadrant (Figure 1C (Fig. 1)). Left-eye fundus autofluorescence was within normal limits with artifact from sub-capsular cataract (Figure 1D (Fig. 1)).

#### B-scan ultrasound

**Figure 2:**
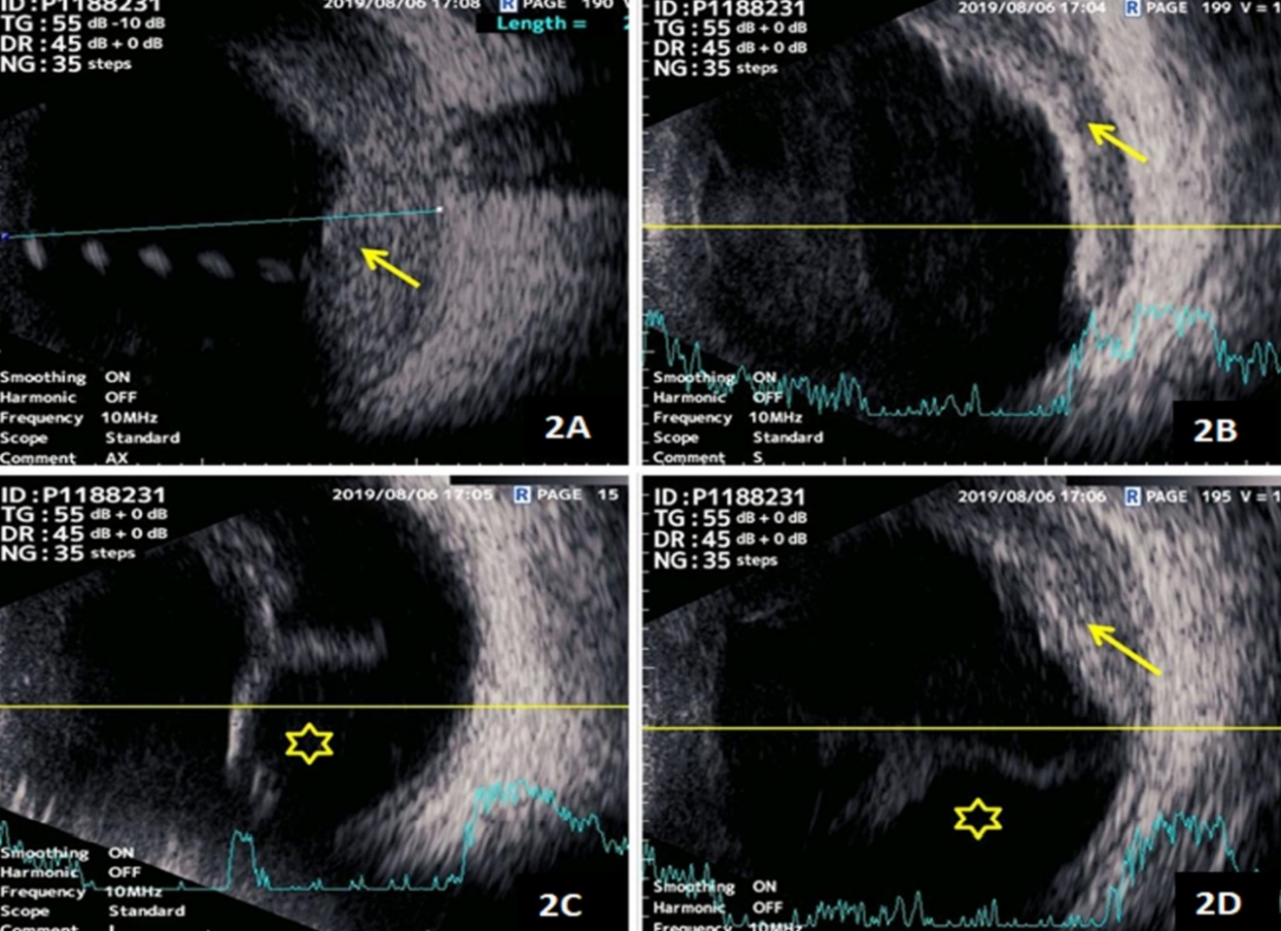
A) Right-eye ultrasound B-scan in the axial scan showed an elevated mass lesion in the choroid with moderate to high internal echogenicity (yellow arrow). B–D) The transverse and dynamic ultrasound B-scans also showed presence of exudative retinal detachment in the inferior and nasal scans (yellow asterisk in 2C and 2D) with elevated moderate to high echogenic choroidal lesions in the superior and nasal scans (yellow arrow in 2B and 2D).

Right-eye ultrasound B-scan in the axial scan showed an elevated mass lesion in the choroid with moderate to high internal echogenicity. The transverse and dynamic ultrasound B-scans also showed the presence of an exudative retinal detachment in the inferior scans with elevated choroidal lesions in the superior scans (Figure 2 (Fig. 2)). 

#### Optical coherence tomography (OCT)

**Figure 3:**
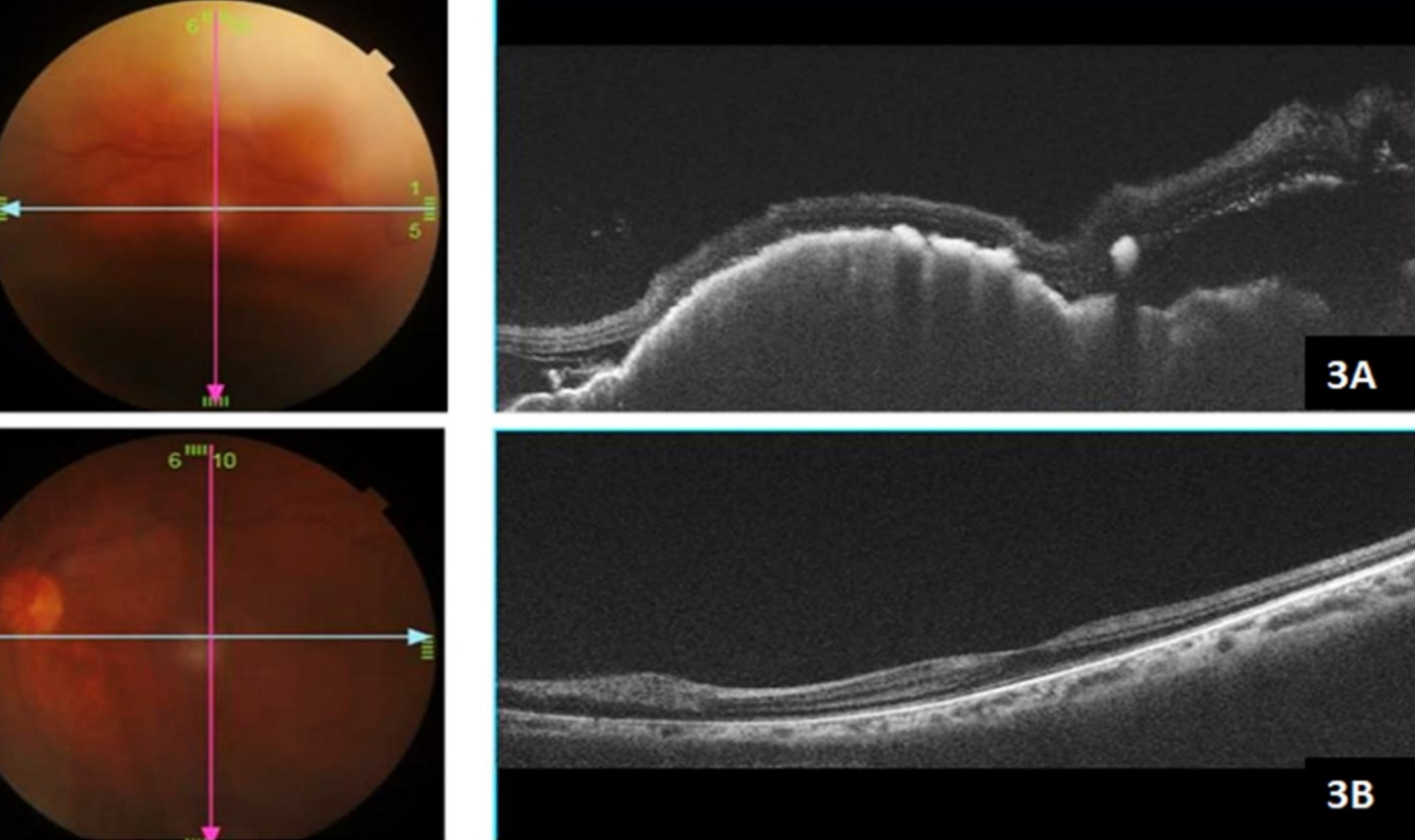
A) Right-eye OCT line scan passing through the temporal aspect of the fundus showed altered retinal contour, and a subretinal hyporeflective space with few high reflective lesions subretinally. It also showed lumpy-bumpy appearance of the retinal pigment epithelium and an elevated choroidal mass lesion beneath it. B) The left showed a normal foveal OCT scan.

Optical coherence tomography (OCT) of the right eye was of poor quality due to the presence of exudative retinal detachment and the patient’s inability to fixate due to very poor vision. Right-eye OCT line scan passing through the temporal aspect of the fundus showed altered retinal contour, and a subretinal hyporeflective space with few high reflective lesions subretinally. It also showed a lumpy-bumpy appearance of the retinal pigment epithelium and an elevated choroidal mass lesion beneath it (Figure 3A (Fig. 3)). The left showed a normal foveal OCT scan (Figure 3B (Fig. 3)).

In view of the above findings and considering the old age of the patient, a thorough systemic evaluation was carried out. On evaluation it was found that the patient had a history of significant weight loss, loss of appetite, cough, shortness of breath, enlarged lymph nodes in the neck, and also on-and-off episodes of pain in the abdomen.

#### High-resolution computed tomography (HRCT)

HRCT of the neck and chest showed enlarged tracheal lymph nodes, soft tissue lesions in the lower lobes of the lungs with pleural effusion – features more in favor of bronchogenic carcinoma. The HRCT also showed increased soft tissue density in the right adrenal gland and osteolytic lesions in the lower dorsal vertebra suggestive of metastatic lesions. The above systemic findings and ocular findings led us to the diagnosis of ocular choroidal metastasis secondary to lung cancer with probable bronchogenic carcinoma. The patient was advised for a lung biopsy after consulting with a pulmonologist. She was also referred to an oncologist to initiate the chemotherapy.

### Treatment

Our patient was started on systemic chemotherapy to take care of both primary and metastatic lesions.

### Outcome and follow-up

Our patient was under care with a pulmonologist and an oncologist.

## Discussion

The choroid is the most common site for ocular metastasis from a systemic carcinoma due to its rich blood supply. The most common site of primary causes for ocular metastasis is the breast, followed by the lung [1], [2]. It has been reported that the incidence of choroidal metastasis from breast cancer is 37–41%, and from the lung 7% [3]. The patients usually present with one or more symptoms of blurred vision, floaters , flashes, and defective field of vision [4]. A good clinical suspicion with thorough systemic evaluation is needed to diagnose ocular metastasis. In most cases, the choroidal metastatic lesions regress with systemic therapy alone [5]. However, for non-responding cases, intravitreal chemotherapy or antivascular endothelial growth factors are also suggested [6]. It has also been shown that ocular radiotherapy is useful in non-regressing ocular metastatic lesions as well [7]. Our case highlights the importance of the ophthalmologist’s role in diagnosing a life-threatening primary malignancy. We would also like to highlight the importance of a regular annual eye check-up of every patient, especially in older age groups, for an early detection of many asymptomatic conditions, for example as in diabetic retinopathy, hypertensive retinopathy, age-related macular degeneration, or metastatic lesions. An early detection of any malignancy will add to the quality of life years of patients.

## Conclusion

1. Choroidal metastasis can be a presenting feature in malignancies of for example the breast or the lung.
2. A thorough systemic evaluation to rule out malignancies elsewhere in the body is to be carried out in case of any unusual presentation in ophthalmology.
3. Our case also highlights the role of the ophthalmologist in diagnosing a life-threatening primary malignancy.
4. An early detection of any malignancy will add to the quality of life years of patients.

## Notes

### Competing interests

The authors declare that they have no competing interests.
